# Neurodegenerative disorders: the Glia way forward

**DOI:** 10.3389/fphar.2014.00157

**Published:** 2014-07-15

**Authors:** Roger A. Barker, Francesca Cicchetti

**Affiliations:** ^1^Department of Clinical Neuroscience, John van Geest Centre for Brain Repair, University of CambridgeCambridge, UK; ^2^Axe Neurosciences, Department of Psychiatry and Neurosciences, Centre de Recherche du CHU de Québec, Université LavalQuébec, QC, Canada

**Keywords:** immunity, inflammation, neurodegenerative disorders, therapeutics, animal models

The realization that inflammation can affect the brain is not a new one, and many diseases of the CNS, such as multiple sclerosis (MS), have been shown to clearly respond to agents that target the immune system at one level or another. However, it is only more recently that the immune system has been shown to play a role in the normal development and homeostasis of the brain as well as contributing to disorders that have traditionally been thought of as being purely neurodegenerative in nature, such as Huntington's (HD) or Alzheimer's disease (AD). In this special issue of Frontiers in Neuropharmacology, we have sought to bring together experts in this new emerging area of neurobiology.

The best place to start in this special issue is with the paper by McGeer and McGeer (2011) as they lay out the history of the field and the skepticism that it has generated en route, and which still exists today. Indeed, many neurologists and neurobiologists still argue that any inflammatory or glial response in neurodegenerative disorders is simply a secondary phenomenon of no pathogenic or therapeutic relevance. However, evidence has accumulated in favor of it having a role and part of this relates to our ability to better image the microglial responses in patients with neurodegenerative disorders of the brain and Politis et al. (2012) explore this in their review on PET and TSPO radioligands.

A number of our papers summarize the role of microglia in ongoing CNS activities. Ekdahl (2012), for example, discusses how microglia may regulate adult neurogenesis in the healthy and diseased brain possibly through direct synaptic contacts. This is taken on by Streit and Xue (2012) who put forward the theory that the loss of the normal neuroprotective function of microglial, as a result of aging, leads to disease states, especially AD. Neher et al. (2012), on the other hand, discuss how microglia can directly phagocytose apparently healthy neurons as well as those in the diseased brain and by so doing are primary players in the cell loss seen in neurodegenerative disorders. This is explored in more detail by Phani et al. (2012) in Amyotrophic Lateral Sclerosis (ALS); Solito and Sastre (2012) in AD; and Huang and Halliday (2012) for Parkinson's disease (PD).

Finally in the first of our two reviews, we discuss how the astrocytic and microglial responses are different in a range of new experimental therapies for neurodegenerative disorders. These therapies include chronic deep brain stimulation, which induces a variable astro- and microglial response and in some cases even a collagenous band at the electrode tip. In contrast, growth factor infusions and gene therapies produce much less of a glial response whilst neural grafts vary in the intensity of response they provoke, in part as a function of disease state. The significance of these different responses (the type and magnitude) remains unknown, but what is emerging from these studies is the complex interplay that exists between glial cells and neurons and how astrocytes, in particular, can influence the activity of large neural and blood networks (Cicchetti and Barker, 2014). These new observations will likely help explain the variance seen in clinical trials using these agents in a range of neurodegenerative disorders.

In our second review we summarize the major findings of the papers that make up this special edition and how we can build on this work as we move forward into a new therapeutic era, which includes the use of a whole new range of immune modulating therapies (Barker and Cicchetti, 2012).

## Conflict of interest statement

The authors declare that the research was conducted in the absence of any commercial or financial relationships that could be construed as a potential conflict of interest.
